# 2018 outstanding contributions to ISCB award: Russ Altman

**DOI:** 10.1371/journal.pcbi.1006140

**Published:** 2018-05-17

**Authors:** Christiana N. Fogg, Diane E. Kovats, Ron Shamir

**Affiliations:** 1 Freelance Writer, Kensington, Maryland, United States of America; 2 International Society for Computational Biology; 3 Blavatnik School of Computer Science, Tel Aviv University, Tel Aviv, Israel

**Figure:**
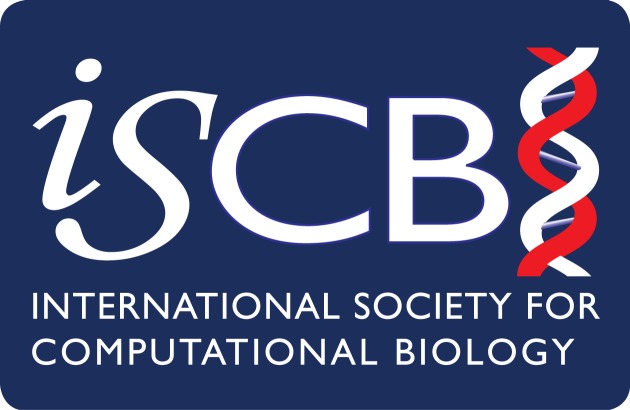


**Image 1:**
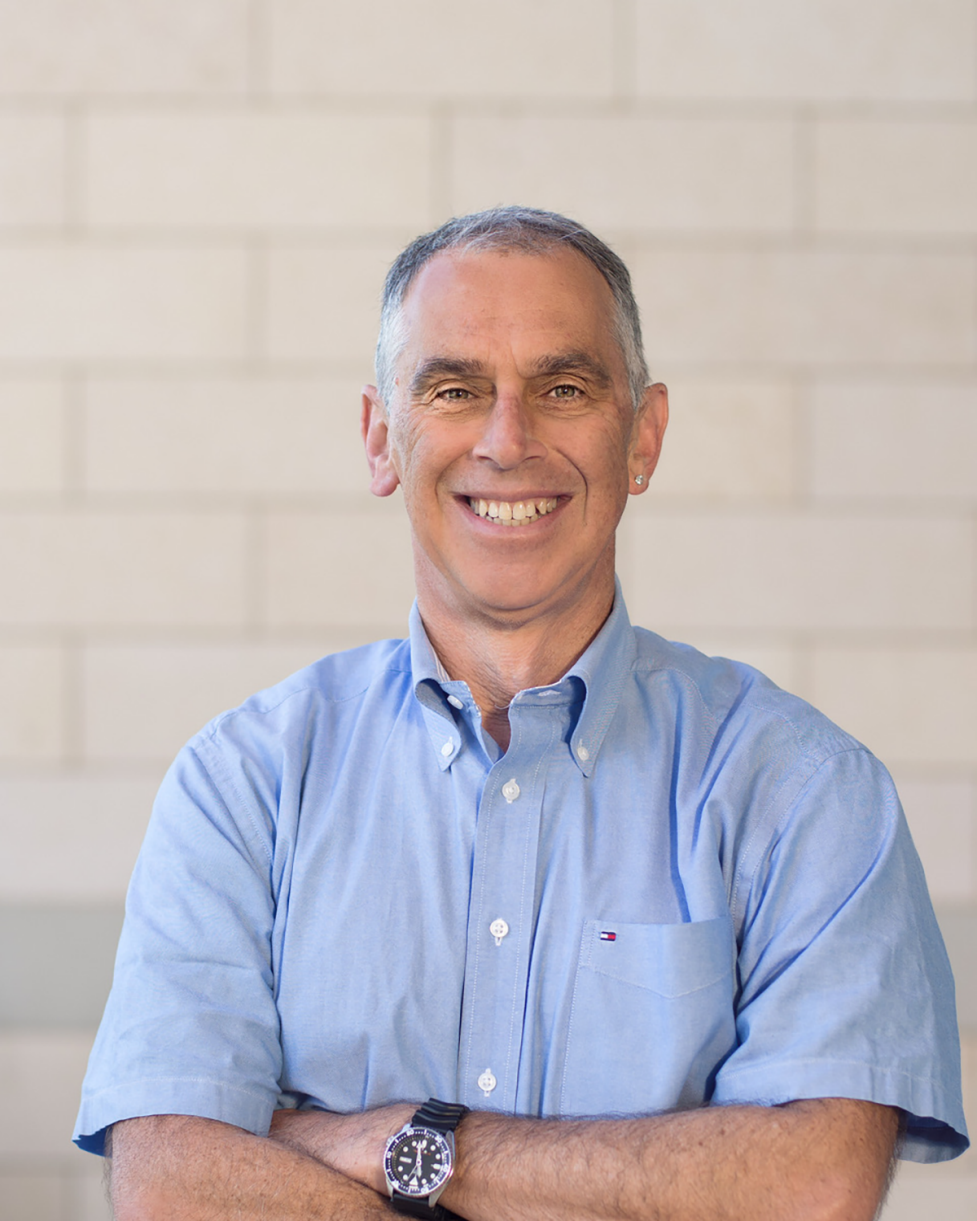
Russ Altman, Kenneth Fong Professor and Professor of Bioengineering, of Genetics, of Medicine (General Medicine Discipline), of Biomedical Data Science, and, by courtesy, of Computer Science.

The Outstanding Contributions to ISCB Award was introduced in 2015 to recognize Society members who have made lasting and beneficial contributions through their leadership, service, and educational work, or a combination of these areas. Russ Altman (Image 1), Kenneth Fong Professor and Professor of Bioengineering, of Genetics, of Medicine (General Medicine Discipline), of Biomedical Data Science, and, by courtesy, of Computer Science, is the 2018 winner of the Outstanding Contributions to ISCB Award and will be recognized at the 2018 Intelligent Systems for Molecular Biology (ISMB) meeting in Chicago, Illinois, being held on July 6–10, 2018.

Altman’s years of dedicated service to ISCB began when he attended the very first ISMB meeting in 1993. As a brand new faculty member, he remembered how he felt at home at ISMB, surrounded by a community of scientists also interested in computational biology and bioinformatics. Altman’s enthusiasm at this first ISMB meeting led him to help organize the next ISMB meeting. He recalled, “It became clear that there was no obvious ‘host’ for ISMB 1994, so I volunteered to host it at Stanford, where we had a lovely meeting with a couple of hundred people. We had some extra money after paying our bills, so we wanted to send the money to wherever ISMB 1995 was going to be (UK). For the first few years, this is how ISMB worked—the organizers from one year would send the leftover funds as a seed for the next ISMB. There was no organization, and as the size of the leftover check increased, we started getting nervous and realized we needed to create a legal entity.” ISCB was born at ISMB 1997 in Halkidiki, Greece, where organizers of former ISMB meetings and others sat at dinner on the beach and planned the society and figured out how to incorporate it. Altman has warm recollections of that historic gathering and said, “There are pictures of that great dinner and group, and I treasure the memory of that meeting”.

Altman has enjoyed serving ISCB at all levels since its inception, from work on the Publications Committee and as a conference organizer to his tenure on the ISCB Board of Directors (1997–2005) and as ISCB President (2002–2005). Altman’s early work on the Publications Committee included applying for PubMED to index the ISMB proceedings, which was a critical step in helping ISCB members receive academic credit for their conference papers. Altman also helped negotiate the agreement to have *Bioinformatics* named as an official ISCB journal. Beyond ISMB, Altman has been an organizer of the Pacific Symposium on Biocomputing and has facilitated the relationship between this conference and ISCB.

As computational biology and bioinformatics have grown into stand-alone fields, Altman has made many critical scientific contributions through his research. Altman and his research group have developed numerous computational tools that address problems in basic biology and medicine, with a particular interest in understanding drug responses. His work has included studies of structure–function relationships in macromolecules, understanding RNA structure and folding, and assessing drug responses at the molecular, cellular, organismal, and population levels.

Altman believes that it is critical to bring awareness to the greater scientific community that computational biologists and bioinformaticians are more than just great collaborators, but they also lead major research projects. He considers service to ISCB as a way established PIs, junior faculty, and trainees can help bring about this awareness to advance the field. Altman considers ISCB to be a community that provides both valuable service opportunities and sources of mentorship and collaboration for scientists.

Altman’s dedication to the field of computational biology has been recognized by his election as an ISCB Fellow (2010), as well as with numerous other honors, including election as a member of the National Academy of Medicine (formerly the Institute of Medicine, 2009) and a Fellow of the American Association for the Advancement of Science (2014). Altman has also worked as an editor and reviewer for numerous scientific journals, including serving as Co-Editor-in-Chief of the Annual Review of Biomedical Data Science.

Altman’s many years of service to ISCB have been critical to the very formation and evolution of the Society from its infancy as a small meeting to the globally recognized professional organization that it is today.

